# World Hip Trauma Evaluation (WHiTE): framework for embedded comprehensive cohort studies

**DOI:** 10.1136/bmjopen-2016-011679

**Published:** 2016-10-21

**Authors:** Matthew L Costa, Xavier L Griffin, Juul Achten, David Metcalfe, Andrew Judge, Rafael Pinedo-Villanueva, Nicholas Parsons

**Affiliations:** 1 NDORMS (Nuffield Department of Orthopaedics, Rheumatology, and Musculoskeletal Sciences), Kadoorie Centre, University of Oxford, John Radcliffe Hospital, Oxford, UK; 2 NDORMS (Nuffield Department of Orthopaedics, Rheumatology, and Musculoskeletal Sciences), University of Oxford, Oxford, UK; 3 Warwick Medical School, University of Warwick, Coventry, UK

**Keywords:** ORTHOPAEDIC & TRAUMA SURGERY, TRAUMA MANAGEMENT

## Abstract

**Introduction:**

Osteoporotic hip fractures present a significant global challenge to patients, clinicians and healthcare systems. It is estimated that hip fracture accounts for 1.4% of total social and healthcare costs in the established market economies.

**Methods and analysis:**

The World Hip Trauma Evaluation (WHiTE) was set up to measure outcome in a comprehensive cohort of UK patients with hip fracture. All patients in the cohort are treated under a single comprehensive treatment pathway. A core outcome set, including health-related quality of life, is collected on all the patients. This protocol describes the current multicentre project that will be used as a vehicle to deliver a series of embedded observational studies.

**Ethics and dissemination:**

Research Ethics Committee approval was granted (Rec reference 11/LO/0927, approved 18/8/2011) and each hospital trust provided National Health Service (NHS) approvals.

**Trial registration number:**

The study is registered with National Institute of Health Research Portfolio (UKCRN ID 12351) and the ISRCTN registry (ISRCTN63982700).

## Introduction

The World Hip Trauma Evaluation (WHiTE) project was set up to measure outcome in a comprehensive cohort of UK patients with hip fracture. All patients in the cohort are treated under a single comprehensive treatment pathway. A core outcome set, including health-related quality of life (QoL), is collected on all the patients.

This protocol describes the current multicentre project that will be used as a vehicle to deliver a series of embedded observational studies.

Osteoporotic hip fractures present a significant global challenge to patients, clinicians and healthcare systems. It is estimated that hip fracture accounts for 1.4% of total social and healthcare costs in the established market economies.^1^ Approximately 8.5% of patients with hip fracture will die within 30 days of injury^2^ and 22.5% within 12 months.^2^ ^3^ In addition, the majority of survivors experience a significant reduction in health-related QoL.^4^ ^5^ The challenge is expected to intensify with a rise from the 1.3 million hip fractures in 1990 to 6.3 million globally by 2050.^6^

The National Hip Fracture Database (NHFD) already captures data on almost all patients with hip fracture in England, Wales and Northern Ireland.^7^ Although it is used principally for national clinical audit, there is considerable potential to use the NHFD for research purposes.^3^ However, the NHFD does not routinely capture patient-reported outcome measures and there is substantial variation in clinical decision-making (eg, choice of fixation device) between hospitals and individual surgeons, which risks introducing confounding to observational studies embedded within the NHFD.^8^

In order to address these limitations, we established the Warwick Hip Trauma Evaluation (WHiTE) study to measure outcome in a comprehensive cohort of hip fracture patients within the framework of the NHFD.^9–13^ This study was renamed ‘World Hip Trauma Evaluation’ following the decision to make the project multicentre in 2015. The WHiTE project is nested within the NHFD and all patients are treated under a single comprehensive treatment pathway that is based on the National Institute for Health and Care Excellence (NICE) Hip Fracture Guidelines.^14^ This should help limit unexplained variability in clinical decision-making. The WHiTE project will also enrich the NHFD data by collecting patient-reported outcome measures and functional status after discharge from hospital. These are included within a ‘core outcome set’ that has been established for this patient group; both outcome measures and data collection systems were tested in a series of single-centre pilot investigations.^11–13^

As WHiTE is nested within a clinical registry, it will capture variables (eg, fracture pattern and operation type) that are particularly important for hip fracture research. The data are also collected prospectively by dedicated research associates at each hospital site for the principle purpose of research. It will therefore provide a richer resource for hip fracture research than existing UK administrative data sets, such as Hospital Episode Statistics (HES) and the Clinical Practice Research Datalink (CPRD).

### Objective

The objective of the WHiTE project is to provide a data collection framework in which a series of observational studies can be embedded by hip fracture researchers.

## Patients and methods

### Eligibility

All patients presenting with a hip fracture to one of the recruiting National Health Service (NHS) sites and who would currently be reported to the NHFD. Alignment with the NHFD eligibility criteria will reduce the data collection burden at recruiting sites, which is important for its sustainability as further studies are embedded within the WHiTE framework. The NHFD exclusion criteria are:

- Age <60 years.
- Non-operative treatment.^7^

Throughout the study, screening logs will be kept to determine the number of patients assessed for eligibility and reasons for any exclusion. Patients who decline to consent to take part will be given the opportunity to inform the research team of the reasoning behind their decision not to participate.

### Consent

Patients with a hip fracture are a clinical priority for urgent operative care and will usually undergo surgery on the next available trauma operating list. All WHiTE recruiting sites treat hip fracture patients according to national guidelines and so patients will be managed according to a series of common standard treatment pathways. The WHiTE study will not affect their treatment in any way. The patients will be approached for consent to be part of this cohort study in the immediate postoperative period.

At the first appropriate time when the patient has regained capacity (usually the first day after surgery) a research associate will provide the participant with the study information. The patient will be given the opportunity to ask questions and discuss the study with their family and carers for as long as they require. They will then be asked to provide written consent to participate in the study. If the patient does not wish to complete questionnaires for the study at this stage, they will be asked if they are happy to provide written consent for us to access and use any routine NHS data, including that collected through the NHFD. Alternatively the patient can choose to decline to have any data entered into the study.

Best efforts will be made to involve participants who, temporarily or permanently, lack capacity in the decision to be involved in the study. The clinical team will make a judgement about the amount and complexity of the information that the participant is able to understand and retain on an individual basis. Appropriate information will be communicated to the participant and updated as their understanding changes. In the event that a patient has temporary loss of capacity, we will follow best practice and ask the patient for consent as soon as they are able to do so.

In accordance with section 32 subsection 9b of the Mental Capacity Act 2005, where a patient does not have capacity to consent for themselves, we will seek agreement from an appropriate consultee.

Where a personal consultee is available, they will be provided with the study information. The personal consultee will be given the opportunity to ask questions and discuss the study after which they will be asked to provide agreement, which will be documented. The personal consultee will complete the outcome questionnaires on behalf of the patient.

Where a personal consultee is not available, a nominated consultee will be identified to advise the research team. This person will be the patient's treating surgeon and/or their treating general practitioner. If their surgeon is a member of the research team, another independent surgeon will be identified.

If the patient withdraws from the study, data collected up until the point of withdrawal will be included in any subsequent analyses.

At all times the chief investigator will act in accordance with the patients' best interests. Any new information that arises during the trial that may affect participants' willingness to take part will be reviewed by the Oversight Committee; if necessary this will be communicated to all participants and a revised consent form completed.

### Standardised treatment pathway

Participants will be treated in accordance with a standardised care pathway from diagnosis to final discharge from hospital. This pathway is described below and is in accordance with national guidance issued by NICE.^14^

*Preoperative assessments*: Participants will usually be assessed in the emergency department. Diagnosis of a fracture of the hip will be confirmed by appropriate radiographic images. Where there is doubt over the presence or the radiological pattern of the fracture, further imaging will be performed as per routine clinical practice.

All participants will have initial assessment as part of a multidisciplinary pathway and will undergo the following investigations as a minimum: ECG, full blood count, group and save, and tests of renal function. A cognitive assessment will be undertaken.

Routine thromboprophylaxis will be started in all participants not already receiving anticoagulant therapy. Chemical and mechanical prophylaxis measures will be used in accordance with current protocols agreed at each centre.

*Anaesthetic technique*: A regional or general anaesthesia technique will be used for every participant. The anaesthetic procedure may incorporate a local anaesthetic nerve block using either a nerve stimulator or ultrasound-guided technique or periarticular analgesic injections in accordance with local protocols.

*Fracture pattern*: Fracture patterns will be classified preoperatively from the radiographs into four groups:

- Group 1: undisplaced/valgus impacted intracapsular;
- Group 2: displaced intracapsular;
- Group 3: trochanteric—fractures centred within the trochanteric region;
- Group 4: subtrochanteric—fractures centred within the region 5 cm below the lesser trochanter.

*Operative intervention*: All participants should aim to have surgery on the day or the day after injury on a consultant-led operating list. All participants will receive perioperative prophylactic antibiotics in accordance with current protocols agreed at each centre.

- *Group 1: undisplaced intracapsular fractures*: all participants will have their fracture fixed in situ. Internal fixation of the fracture will be achieved through a standard mini open lateral or percutaneous approach with parallel cannulated screws.
- *Group 2: displaced intracapsular fractures*: the operating surgeon will perform their preferred approach. The hip will be dislocated and the head excised. There are two subgroups:
  The majority of patients with low prefracture functional demand—a proven hemiarthroplasty femoral component will be cemented into the femur using a third generation cementing technique.
  A subgroup of patients with high prefracture functional demand—total hip replacement (THR), where the acetabulum is also replaced. Given the lack of evidence to support criteria to select those participants who might benefit from THR a pragmatic approach will be adopted. Eligibility for this subgroup will be determined by the treating orthopaedic surgeon, on the basis that he or she believes the participant would benefit from total hip arthroplasty. In the event of a participant undergoing THR the approach, implant and operative technique employed would be at the discretion of the operating surgeon.
- *Group 3: trochanteric fractures*: All participants will undergo reduction of their fracture on a fracture table. There are three subgroups:
  Stable standard-obliquity fractures (AO/OTA A1)—internal fixation with a sliding hip screw.
  Unstable standard-obliquity fractures (AO/OTA A2)—internal fixation using a sliding hip screw.
  Unstable reverse obliquity or fractures at the level of the lesser trochanter (AO/OTA A3)—internal fixation with a distally locked, cephalomedullary device.
- *Group 4: subtrochanteric fractures*: All participants will undergo reduction of their fracture on a fracture table. Internal fixation of the fracture will be achieved using a distally locked, cephalomedullary device.

*Postoperative rehabilitation*: Post-operative analgesia will be prescribed perioperatively and reviewed by the responsible clinical teams as appropriate. In the postoperative period, participants will undergo an initial physiotherapy and occupational therapy trauma assessment. A full social, cognitive, premorbid function and falls history will be obtained and documented. An initial rehabilitation plan will be followed with all participants. All patients will undergo both a bone health assessment and falls risk assessment as per usual care.

*Discharge from hospital*: Participants will be discharged from the acute orthopaedic trauma ward at the earliest safe opportunity to the most appropriate discharge destination as determined by the multidisciplinary team.

### Outcome data

In this study, we will use techniques common in long-term cohort studies to ensure minimum loss to follow-up.

We will attempt to contact the patient or next of kin by telephone. If this fails we will send the patient or next of kin a postal questionnaire to complete with a prepaid return envelope. Finally the general practitioners of those participants who are deemed ‘lost to follow-up’ will be contacted in order to attempt to complete the follow-up. If all these methods fail, then we will class the patient as a non-responder for that time point.

*Health-related QoL score (EQ-5D)*: EuroQol (EQ-5D-5L) at 4 months postinjury, adjusted for preinjury score. The EQ-5D-5L is a validated self-administered patient-reported outcome measure and requires 5 min to complete. EQ-5D is a generic health-related QoL measure consisting of five dimensions each with a five-level answer possibility. Each combination of answers produces a health profile which can be converted into an estimated health utility score after applying a set of preference weights.^15^ The preliminary English social value set obtained from general population preferences will be used^16^ to estimate health utilities until one for the UK is produced and made available. The EQ-5D has good test–retest reliability, is simple for patients to use, and gives a single preference-based index value for health status that can be used for broader cost-effectiveness comparative purposes.

*Residential status*: Prefracture and current residential status will be collected at 4 months. The possible responses for residential status are: own home/sheltered housing, residential care, nursing home, rehabilitation unit, acute hospital, dead, other.

*Mobility status*: Prefracture and current mobility status will be collected at 4 months. The possible responses for mobility status are: freely mobile without aids, mobile outdoors with one aid, mobile outdoors with two aids or frame, some indoor mobility but never goes outside without help, no functional mobility (using lower limbs).

*Hospital information*: *Admission* including admission dates and orthopaedic/orthogeriatrician details. *Assessment* including Abbreviated Mental Test Score, pathological fracture and preoperative medical assessment information along with fracture classification and side. *Treatment* including operation details. *Discharge* including discharge summary.

*Complications*: The complications that we will report as ‘expected’ following hip fracture are: wound infection, respiratory infection, urinary tract infection, venous thromboembolism, cerebrovascular accident, cardiac event, failure of fixation, dislocation, blood transfusion.

### End of study

All patients will be followed up to collect data on their status at 4 months postoperation. If contact cannot be made or data collected within 3 months of this point, follow-up will not be collected. After the 4-month follow-up, participants will be treated as per normal standard of care with no further study-specific follow-up.

### Data collection and data management

Personal data collected during the trial will be handled and stored in accordance with the 1998 Data Protection Act and CTU Standard Operating Procedures.

The data collected from participants will be entered in the study and NHFD databases. All data collected will be anonymised after the collection of the baseline demographic data and all participants given a unique study number. Identifiable participant data will be held on a separate database and in a locked filing cabinet and coded with a study participant number to tag identifiable data to the outcome data.

With the patient's consent, data may be linked to routine NHS and social care data sets via the Health and Social Care Information Centre or appropriate body.

Disclosure of confidential information will only be given if a participant indicates an issue that may jeopardise the safety of the participant or another person.

### Oversight Committee

The Oversight Committee comprises of representatives from each of the professional groups involved in the care of patients with hip fracture and lay representatives of the patient group. They will collectively manage the data governance arrangements, although final responsibility will lie with the chief investigator.

## Data analysis

### Observational studies

The WHiTE framework will be suitable for addressing a broad range of hip fracture research questions, either alone or in concert with national data from its parent registry, the NHFD. It will not be suitable for studies relating to younger adults with hip fractures or those treated non-operatively. An illustrative list of the types of research question for which WHiTE may be suitable includes:

*Descriptive epidemiology*: Describing the occurrence and distribution of hip fractures in the UK, for example, is there an association between socioeconomic status and outcome, including loss of health-related QoL, after hip fracture?

*Health services research*: Exploring the effects of differences in healthcare delivery, for example, does hospital hip fracture volume affect patient outcomes?

*Outcome studies*: Studying the impact of treatment decisions on patients, for example, does compliance with NICE guidelines improve outcomes for patients with hip fractures?

The Oversight Committee will prioritise individual study ideas and manage the application process for using WHiTE data. They will prospectively approve study applications in advance of any data being shared or analysed.

### Sample size

A sample size for a study such as the one we propose here is difficult to determine with any degree of precision. This is, in large part, due to the flexibility of the approach being adopted, that is, specific research questions will be identified by the Oversight Committee over time.

However, there are aspects that are determined a priori that can guide an initial sample size. A key determinant of the sample size, and also an important outcome in itself, in this frail (elderly) population is the death rate. Previous analyses^5^ ^13^ have shown that the death rate in this group is between 20% and 30% at 12 months, but data were sparse and limited. One research question that we know will be important is to establish the overall population death rate and model how it changes in the immediate postoperative period. Table 1 shows 95% CIs for estimates of death rates of 20% and 30% for varying sample sizes (n), based on a binomial model.

**Table 1:** CIs (95%) for death rates of 20% and 30% by sample size (n)

	Death rate	
n	20%	30%
100	(12.9 to 29.4)	(21.5 to 40.1)
300	(15.7 to 25.1)	(24.9 to 35.6)
1000	(17.6 to 22.6)	(27.2 to 33.0)
3000	(18.6 to 21.5)	(28.4 to 31.7)
6000	(19.0 to 21.0)	(28.8 to 31.2)

In order to estimate the death rate to a high level of precision (within 1%), a reasonable target overall sample size is therefore ∼6000 participants. The death rate is likely to vary within important subgroups of the population (eg, fracture type), so it is also important to consider the precision of estimation of the death rate within the smallest clinically important subgroup, which we think is likely to be in the order of 5% of the overall population. Based on 6000 participants, this amounts to a subgroup of 300, and a CI of ∼10% for estimating the death rate.

Formal analyses will be undertaken, after identification of important research questions by the Oversight Committee, using multiple regression analyses with QoL (EQ-5D) as the response variable. Therefore, Cohen's f^2^ provides a useful way for characterising the probable effect size when fitting such models for continuous outcomes.^17^ Sticking with the above setting, and the smallest clinically important subgroup (n=300) indicates that we will have 95% and 75% power to detect an effect size of 0.08 and 0.05, respectively, at the 5% level in this population and assuming we use 10 degrees of freedom for model fitting. Typical values of f^2^ of 0.02 and 0.15 represent small and moderate effects sizes, so values in the range 0.05–0.08 are small to moderate. Cohen's f^2^ is determined by the ratio of the proportion of variance accounted for in the multiple regression model (regression R^2^ value) to one minus that proportion. Experience suggests that R^2^ values are often small (10–20%) in the setting described here, as QoL is very variable and much of the variability will not be captured by the small number of explanatory variables we will have available for model fitting. Nevertheless, even if models account for only 10% of the variance in QoL we should still have considerable power (>80%) to address important regression relationships within our subgroups of size 300, even allowing some reduction in this number for loss to follow-up.

In summary, an initial sample size of 6000 patients will provide considerable power to estimate overall population characteristics such as death with a high level of precision. Assuming that we are interested in clinically significant subgroups no smaller than 5% of this total (n=300) we will have good power to detect small to moderate effects sizes in our planned multiple regression analyses. On average, each of the WHiTE recruitment sites treats 500 patients with hip fracture a year (range 300–700; NHFD reports) and so we anticipate reaching the 6000 patient sample in 2017.

It is anticipated that the data generated by the cohort study will be used to support and inform hypotheses that are suggested by clinical practice. The Oversight Committee will decide whether any interim analyses are necessary to inform future questions in order to prevent repeated attempts to interrogate the data set without a priori hypotheses. The cohort study data will also be used to update estimates of ‘nuisance’ parameters, such as the death rate and QoL variability in the study population. These nuisance parameters will be used to refine estimates of sample sizes for future embedded randomised controlled trials.

An example observational study within the WHiTE observational cohort might investigate whether the type of fracture a patient sustains influences the patient's outcome. While the optimal interventions for each fracture pattern are defined in current guidelines, the effect of fracture pattern on patient outcome is not well understood. Data from the NHFD 2014 report (n=64 838) give a breakdown of fracture types as follows: undisplaced intracapsular 9.5% (n=6165), displaced intracapsular 48.8% (n=31 656), trochanteric 34.6% (n=22 422), subtrochanteric 5.4% (n=3507) and not specified 1.7% (n=1088). Suppose we were interested in understanding the relationship between the type of intervention, anaethestic and rehabilitation within the subtrochanteric fracture subgroup. This subgroup comprises ∼5.4% (n=325) of the study population (n=6000). Let us assume 90% follow-up rates at 4 months (ie, n is ∼300), with EQ-5D as the primary outcome, and the definitive analysis is a multiple linear regression adjusting for age and gender as minimum. Taking a conservative estimate of R2^5^ ^13^ to give a value of Cohen's f^2^ of 0.05, suggests that we will have 85% power to detect a statistically significant regression analysis, at the 5% level, in the smallest subgroup of interest (subtrochanteric fractures) for a sample size of 300 patients. A 4-month follow-up rate of >90% is anticipated based on a WHiTE pilot study, which successfully followed up 88% of patients at 12 months.^5^

Analogous economic burden and health economic assessments will be integrated into the analysis plan for each observational study. The economic assessments will be conducted from the recommended NHS and personal social services perspective.

### Validation

Once the initial sample size of 6000 patients has been achieved, we will use other data sets to externally validate the cohort and determine its generalisability. In particular, we will explore the NHFD, HES and the CPRD to determine whether the WHiTE cohort is truly representative (eg, in terms of age structure) of the wider UK hip fracture population. This should help contextualise WHiTE data before they are used to determine research priorities and inform health policy.

### Dissemination and publication

The results of this study will be disseminated to the hip fracture clinical community via presentations at national and international meetings as well as publication in peer-reviewed journals and social media. Patient reports will also be made available to participants and the wider public via newsletters and podcasts. The results of the observational studies will influence the next generation of hip fracture guidelines, for example the UK NICE Hip Fracture Guidelines, and generate hypotheses that can be tested in interventional study designs.
